# Encoding and Decoding Models in Cognitive Electrophysiology

**DOI:** 10.3389/fnsys.2017.00061

**Published:** 2017-09-26

**Authors:** Christopher R. Holdgraf, Jochem W. Rieger, Cristiano Micheli, Stephanie Martin, Robert T. Knight, Frederic E. Theunissen

**Affiliations:** ^1^Department of Psychology, Helen Wills Neuroscience Institute, University of California, Berkeley, Berkeley, CA, United States; ^2^Office of the Vice Chancellor for Research, Berkeley Institute for Data Science, University of California, Berkeley, Berkeley, CA, United States; ^3^Department of Psychology, Carl-von-Ossietzky University, Oldenburg, Germany; ^4^Institut des Sciences Cognitives Marc Jeannerod, Lyon, France; ^5^Defitech Chair in Brain-Machine Interface, Center for Neuroprosthetics, Ecole Polytechnique Fédérale de Lausanne, Lausanne, Switzerland; ^6^Department of Psychology, University of California, Berkeley, Berkeley, CA, United States

**Keywords:** encoding models, decoding models, predictive modeling, tutorials, electrophysiology/evoked potentials, electrocorticography (ECoG), machine learning applied to neuroscience, natural stimuli

## Abstract

Cognitive neuroscience has seen rapid growth in the size and complexity of data recorded from the human brain as well as in the computational tools available to analyze this data. This data explosion has resulted in an increased use of multivariate, model-based methods for asking neuroscience questions, allowing scientists to investigate multiple hypotheses with a single dataset, to use complex, time-varying stimuli, and to study the human brain under more naturalistic conditions. These tools come in the form of “Encoding” models, in which stimulus features are used to model brain activity, and “Decoding” models, in which neural features are used to generated a stimulus output. Here we review the current state of encoding and decoding models in cognitive electrophysiology and provide a practical guide toward conducting experiments and analyses in this emerging field. Our examples focus on using linear models in the study of human language and audition. We show how to calculate auditory receptive fields from natural sounds as well as how to decode neural recordings to predict speech. The paper aims to be a useful tutorial to these approaches, and a practical introduction to using machine learning and applied statistics to build models of neural activity. The data analytic approaches we discuss may also be applied to other sensory modalities, motor systems, and cognitive systems, and we cover some examples in these areas. In addition, a collection of *Jupyter* notebooks is publicly available as a complement to the material covered in this paper, providing code examples and tutorials for predictive modeling in python. The aim is to provide a practical understanding of predictive modeling of human brain data and to propose best-practices in conducting these analyses.

## Background

A fundamental goal of sensory neuroscience is linking patterns of sensory inputs from the world to patterns of signals in the brain, and to relate those sensory neural representations to perception. Widely used feedforward models assume that neural processing for perception utilizes a hierarchy of stimulus representations in which more abstract stimulus features are extracted from lower-level representations, and passed along to subsequent steps in the neural processing pipeline. Much of perceptual neuroscience attempts to uncover intermediate stimulus representations in the brain and to determine how more complex representations can arise from these levels of representation. For example, human speech enters the ears as air pressure waveform, but these are quickly transformed into a set of narrow band neural signals centered on the best frequency of auditory nerve fibers. From these narrow-band filters arise a set of spectro-temporal features characterized by the spectro-temporal receptive fields (STRFs) of auditory neurons in the inferior colliculus, thalamus, and primary auditory cortex (Eggermont, 2001). STRFs refer to the patterns of stimulus power across spectral frequency and time (spectro-temporal features). Complex patterns of spectro-temporal features can be used to detect phonemes, and ultimately abstract semantic concepts (DeWitt and Rauschecker, 2012; Poeppel et al., 2012). It should also be noted that there are considerable feedback pathways that may influence this process (Fritz et al., 2003; Yin et al., 2014).

Cognitive neuroscience has traditionally studied hierarchical brain responses by crafting stimuli that differ along a single dimension of interest (e.g., high- vs. low-frequency, or words vs. non-sense words). This method dates back to Donders, who introduced mental chronometry to psychological research (Donders, 1969). Donders suggested crafting tasks such that they differ in exactly one cognitive process to isolate the differential mental cost of two processes. Following Donders, the researcher contrasts the averaged brain activity evoked by two sets of stimuli assuming that the neural response to these two stimuli/tasks is well-characterized by averaging out the trial-to-trial variability (Pulvermüller et al., 1999). One then performs inferential statistical testing to assess whether the two mean activations differ. While much has been learned about perception using these methods, they have intrinsic shortcomings. Using tightly-controlled stimuli focuses the experiment and its interpretation on a restricted set of questions, inherently limiting the independent variables one may investigate with a single task. This approach is time-consuming, often requiring separate stimuli or experiments in order to study many feature representations and may cause investigators to miss important brain-behavior findings. Moreover, it can lead to artificial task designs in which the experimental manipulation renders the stimulus unlike those encountered in everyday life. For example, contrasting brain activity between two types of stimuli requires many trials with a discrete stimulus onset and offset (e.g., segmented speech) so that evoked neural activity can be calculated, though natural auditory stimuli (e.g., conversational speech) rarely come in this time-segregated manner (Felsen and Dan, 2005; Theunissen and Elie, 2014). In addition, this approach requires a priori hypotheses about the architecture of the cognitive processes in the brain to guide the experimental design. Since these hypotheses are often based on simplified experiments, the results do not readily transfer to more realistic everyday situations.

There has been an increase in techniques that use computationally-heavy analysis in order to increase the complexity or scope of questions that researchers may ask. For example, in cognitive neuroscience the “Multi-voxel pattern analysis” (MVPA) framework utilizes a machine learning technique known as classification to detect condition-dependent differences in patterns of activity across multiple voxels in the fMRI scan (usually within a Region of Interest, or ROI: Norman et al., 2006; Hanke et al., 2009; Varoquaux et al., 2016). MVPA has proven useful in expanding the sensitivity and flexibility of methods for detecting condition-based differences in brain activity. However, it is generally used in conjunction with single-condition based block design that is common in cognitive neuroscience.

An alternative approach studies sensory processes using multivariate methods that allow the researcher to study multiple feature representations using complex, naturalistic stimuli. This approach entails modeling the activity of a neural signal while presenting stimuli varying along multiple continuous stimulus features as seen in the natural world. In this sense, it can be seen as an extension of the MVPA approach that utilizes complex stimuli and provides a more direct model of the relationship between stimulus features and neural activity. Using statistical methods such as regression, one may create an optimal model that represents the combination of elementary stimulus features that are present in the activity of the recorded neural signal. These techniques have become more tractable in recent years with the increase in computing power and the improvement of methods to extract statistical models from empirical data. The benefits over a traditional stimulus-contrast approach include the ability to make predictions about new datasets (Nishimoto et al., 2011), to take a multivariate approach to fitting model weights (Huth et al., 2012), and to use multiple feature representations within a single, complex stimulus set (Di Liberto et al., 2015; Hullett et al., 2016).

These models come in two complementary flavors. The first are called “encoding” models, in which stimulus features are used to predict patterns of brain activity. Encoding models have grown in popularity in fMRI (Naselaris et al., 2011), electrocorticography (Mesgarani et al., 2014), and EEG/MEG (Di Liberto et al., 2015). The second are called “decoding” models, which predict stimulus features using patterns of brain activity (Mesgarani and Chang, 2012; Pasley et al., 2012; Martin et al., 2014). Note that in the case of decoding, “stimulus features” does not necessarily mean a sensory stimulus—it could be an experimental condition or an internal state, though in this paper we use the term “stimulus” or “stimulus features.” Both “encoding” and “decoding” approaches fall under the general approach of predictive modeling, and can often be represented mathematically as either a regression or classification problem.

We begin with a general description of predictive modeling and how it has been used to answer questions about the brain. Next we discuss the major steps in using predictive models to ask questions about the brain, including practical considerations for both encoding and decoding and associated experimental design and stimulus choice considerations. We then highlight areas of research that have proven to be particularly insightful, with the goal of guiding the reader to better understand and implement these tools for testing particular hypotheses in cognitive neuroscience. To facilitate using these methods, we have included a small sample dataset, along with several scripts in the form of *jupyter* notebooks that illustrate how one may construct predictive models of the brain with widely-used packages in Python. These techniques can be run interactively in the cloud as a GitHub repository^1^.

## The predictive modeling framework

**Figure 1:**
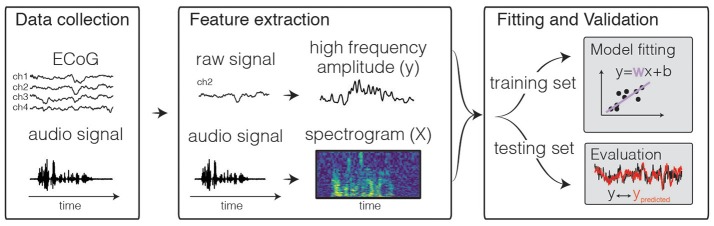
Predictive modeling overview. The general framework of predictive models consists of three steps. First, input and output data are collected, for example during passive listening to natural speech sentences. Next, features are extracted. Traditional features for the neural activity can be the time-varying power of various frequency bins, such as high frequency range (70–150 Hz, shown above). For auditory stimuli, the audio envelope or spectrogram are often used. Finally, the data are split into a training and test set. The training set is used to fit the model, and the test set is used to calculate predictive score of the model.

Predictive models allow one to study the relationship between brain activity and combinations of stimulus features using complex, often naturalistic stimulus sets. They have been described with varying terminology and approaches (Wu et al., 2006; Santoro et al., 2014; Yamins and DiCarlo, 2016), but generally involve the following steps which are outlined below (see Figure 1).

1. **Input feature extraction**: In an encoding model, features of a stimulus (or experimental condition) are used as inputs. These features are computed or derived from “real world” parameters describing the stimulus (e.g., sound pressure waveform in auditory stimuli, contrast at each pixel in visual stimuli). The choice of input features is a key step in the analysis: features must be adapted to the level in the sensory processing stream being studied and multiple feature-spaces can be tried to test different hypotheses. This is generally paired with the assumption that the neural representation of stimulus features becomes increasingly non-linear as one moves along the sensory pathway. For example, if one is fitting a linear model, a feature space based on the raw sound pressure waveform could be used to predict the responses of auditory nerve fibers (Kiang, 1984), but would perform significantly worse in predicting activity of neurons in the inferior colliculus (Andoni and Pollak, 2011) or for ECoG signals recorded from auditory cortex (Pasley et al., 2012). This is because the neural representation of the stimulus is rapidly transformed such that neural activity no longer has a linear relationship with the original raw signal. While a linear model may capture some of this relationship, it will be a poor approximation of the more complex stimulus-response function. At the level of secondary auditory areas, the prediction obtained from higher-level features such as word representations could be contrasted to that based on spectral features (as the alternative feature space) to test the hypothesis that these higher-level features (words) are particularly well-represented in this brain region (de Heer et al., 2017). Other examples of feature spaces for natural auditory signals are modulation frequencies (Mesgarani et al., 2006; Pasley et al., 2012; Santoro et al., 2014), phonemes (Mesgarani et al., 2014; Khalighinejad et al., 2017), or words (Huth et al., 2012, 2016). For stimulus features that are not continuously-varying, but are either “present” or not, one uses a binary vector indicating that feature's state at each moment in time. It may also be possible to combine multiple feature representations with a single model, though care must be taken account for the increased complexity of the model and for dependencies between features (Lescroart et al., 2015; de Heer et al., 2017).
2. **Output feature extraction**: Similarly, a representation of the neural signal is chosen as an output of the encoding model. This output feature is often a derivation of the “raw” signal recorded from the brain, such as amplitude in a frequency band of the time-varying voltage of an ECoG signal (Pasley et al., 2012; Mesgarani et al., 2014; Holdgraf et al., 2016), pixel intensity in fMRI (Naselaris et al., 2011), and spike rates in a given window or spike patterns from single unit recordings (Fritz et al., 2003; Theunissen and Elie, 2014). Choosing a particular *region* of the brain from which to record can also be considered a kind of “feature selection” step. In either case, the choice of features underlies assumptions about how information is represented in the neural responses. In combination with the choice of derivations of the raw signal to use, as well as which brain regions to use in the modeling process, the predictive framework approach can be used to test how and where a given stimulus feature is represented. For example, the assumption that sensory representations are hierarchically organized in the brain (Felleman and Van Essen, 1991) can be tested directly.
3. **Model architecture and estimation**: A model is chosen to map input stimulus features to patterns of activity in a neural signal. The structure and complexity of the model will determine the kind of relationships that can be represented between input and output features. For example, a linear encoding model can only find a linear relationship between input feature values and brain activity, and as such it is necessary to choose features that are carefully selected. A non-linear model may be able to uncover a more complex relationship between the raw stimulus and the brain activity, though it may be more difficult to interpret, will require more data, and still may not adequately capture the actual non-linear relationship between inputs and outputs (Eggermont et al., 1983; Paninski, 2003; Sahani and Linden, 2003; Ahrens et al., 2008). In cognitive neuroscience it is common to use a linear model architecture in which outputs are a weighted sum of input features. Non-linear relationships between the brain and the raw stimulus are explicitly incorporated into the model in the choice of input and output feature representations (e.g., performing a Gabor wavelet decomposition followed by calculating the envelope of each output is a non-linear expansion of the input signal). Once the inputs/outputs as well we the model architecture have been specified, the model is fit (in the linear case, the input weights are calculated) by minimizing a metric of error between the model prediction and the data used to fit the model. The metric of error can be rigorously determined based on statistical theory (such as maximum likelihood) and a probability model for the non-deterministic fraction of the response (the noise). For example, if one assumes the response noise is normally distributed, a maximum likelihood approach yields the sum of squared errors as an error metric. Various analytical and numerical methods are then used to minimize the error metric and, by doing so, estimate the model parameters (Wu et al., 2006; Hastie et al., 2009; Naselaris et al., 2011).
4. **Validation**: Once model parameters have been estimated, the model is validated with data which were not used in the fit: in order to draw conclusions from the model, it must generalize to new data. This means that it must be able to predict new patterns of data that have never been used in the original model estimation. This may be done on a “held-out” set of data that was collected using the same experimental task, or on a new kind of task that is hypothesized to drive the neural system in a similar manner. In the case of regression with normally distributed noise, the variance explained by the model on cross-validated data can be compared to the variance that could be explained based on differences between single data trials and the average response across multiple repetitions of the same trial. This ratio fully quantifies the goodness of fit of the model. While this can be difficult to estimate, it allows one to calculate an “upper bound” on the expected model performance and can be used to more accurately gauge the quality of a model, see section What Is a “Good” Model Score? (Sahani and Linden, 2003; Hsu et al., 2004).
5. **Inspection and Interpretation**: If an encoding model is able to predict novel patterns of data, then one may further inspect the model parameters to gain insight into the relationship between brain activity and stimulus features. In the case of linear models, model parameters have a relatively straightforward definition—each parameter's weight is the amount the output would be expected to change given a unit increase in that parameter's value. Model parameters can then be compared across brain regions or across subjects (Hullett et al., 2016; Huth et al., 2016). It is also possible to inspect models by assessing their ability to generalize their predictions to new kinds of data. See section Interpreting the Model.

This predictive modeling framework affords many benefits, making it possible to study brain activity in response to complex “natural” stimuli, reducing the need for separate experiments for each stimulus feature of interest, and loosening the requirement that stimuli have clear-cut onsets and offsets. Moreover, naturalistic stimuli are better-matched to the sensory statistics of the environment in which the target organism of study has evolved, leading to more generalizable and behaviorally-relevant conclusions.

In addition, because a formal model describes a quantifiable means of transforming input values into output values, it can be “tested” in order to confirm that the relationship found between inputs/outputs generalizes to new data. Given a set of weights that have been previously fit to data, it is possible to calculate the “predictive power” for a given set of features and model weights. This is a reflection of the error in predictions of the model, that is, the difference between predicted outputs and actual outputs (also called the “prediction score”).

While the underlying math is the same between encoding and decoding models when using regression, the interpretation and nature of model fitting differs between the two. The next section describes the unique properties of each approach to modeling neural activity.

### Encoding models

Encoding models are useful for exploring multiple levels of abstraction within a complex stimulus, and investigating how each affects activity in the brain. For example, natural speech is a continuous stream of sound with a hierarchy of complex information embedded within it (Hickok and Small, 2015). A single speech utterance contains many representations of information, such as spectrotemporal features, phonemes, prosody, words, and semantics. The neural signal is a continuous response to this input with multiple embedded streams of information in it due to recording the activity from many neurons spread across a relatively large region of cortex. The components of the neural signal operate on many timescales [e.g., responding to the slow fluctuations of the speech envelope vs. fast fluctuations of spectral content of speech (David and Shamma, 2013)] as information propagates throughout auditory cortex, and are not well-described by a single event-related response to a stimulus onset (Khalighinejad et al., 2017). Naturalistic stimuli pose a challenge for event-related analysis, but are naturally handled in a predictive modeling framework. In the predictive modeling approach, the solution takes the form of a linear regression problem. Hastie et al. (2009)

$$\begin{array}{l} \text{a}ctivit\text{y}\left(t\right)=\overset{N_{features}}{\underset{i}{\sum }}feature_{i}{\left(t\right)}^{*}weight_{i}+error\left(t\right) \end{array}$$

Where the neural activity at time *t* is modeled as a weighted sum of *N* stimulus features. Note that it becomes clear from this equation that features that have never been presented will not enter the model and contribute to the sum. Thus, both the choice of stimuli and input feature space are critical and have a strong influence on the interpretation of the encoding model. It is also common to include several time-lagged versions of each feature as well, accounting for the fact that the neural signal may respond to particular feature patterns in time. In this case, the model formulation becomes:

$$\begin{array}{l} \text{a}ctivity\left(t\right)=\overset{N_{lags}}{\underset{j}{\sum }}\overset{N_{features}}{\underset{i}{\sum }}feature_{i}{\left(t-j\right)}^{*}weight_{i,j}+error\left(t\right) \end{array}$$

In other words, this model describes how dynamic stimulus features are *encoded* into patterns of neural activity. It is convenient to write this in linear algebra terms:

$$\begin{array}{l} activity=Sw+\epsilon \end{array}$$

In this case ***S*** is the stimulus matrix where each row corresponds to a timepoint of the response, and the columns are the feature values at that timepoint and time-lag (there are ${N_{lags}}^{*}N_{features}$ columns). ***w*** is a vector of model weights (one for each feature ^*^ time lag), and **ϵ** is a vector of random noise at each timepoint (most often to be Gaussian for continuous signals or Poisson for discrete signals). The observed output activity can then be written as a single dot product assumed between feature values and their weights plus additive noise. This dot product operation is identical to explicitly looping over features and time lags separately (each “iteration” over lag/feature combinations becomes a column in ***S*** and a single value in ***w***, thus the dot-product achieves the same result).

As mentioned above, the details of neural activity under study (the output features), as well as the input features used to predict that activity, can be flexibly changed, often using the same experimental data. In this manner, one may construct and test many hypotheses about the kinds of features that elicit brain activity. For example to explore the neural response to spectro-temporal features, one may use a spectrogram of audio as input to the model (Eggermont et al., 1983; Sen et al., 2001). To explore the relationship between the overall energy of the incoming auditory signal (regardless of spectral content) and neural activity, one may probe the correlation between neural activity and the speech envelope (Zion Golumbic et al., 2013). To explore the response to speech features such as phonemes, audio may be converted into a collection of binary phoneme features, with each feature representing the presence of a single phoneme (Leonard et al., 2015; de Heer et al., 2017). Each of these stimulus feature representations may predict activity in a different region of the brain. Researchers have also used non-linearities to explore different hypotheses about more complex relationships between inputs and neural activity, see section Choosing a Modeling Framework.

In summary, encoding models of sensory cortex attempt to model cortical activity as a function of stimulus features. These features may be complex and applied to “naturalistic” stimuli allowing one to study the brain under conditions observed in the real world. This provides a flexible framework for estimating the neural tuning to particular features, and assessing the quality of a feature set for predicting brain activity.

### Decoding models

Conversely, decoding models allow the researcher to use brain activity to infer the stimulus and/or experimental properties that were most likely present at each moment in time.

$$\begin{array}{l} \text{feature}\left(t\right)=\overset{N_{lags}}{\underset{j}{\sum }}\overset{N_{channels}}{\underset{i}{\sum }}activity_{i}{\left(t+j\right)}^{*}weight_{i,j}+error\left(t\right) \end{array}$$

which, in vector notation, is represented as the following:

$$\begin{array}{l} s=Xw+\epsilon \end{array}$$

where ***s*** is a vector of stimulus feature values recorded over time, and ***X*** is the channel activity matrix where each row is a timepoint and each column is a neural feature (with time-lags being treated as a separate column each). ***w*** is a vector of model weights (one for each neural feature ^*^ time lag), and **ϵ** is a vector of random noise at each timepoint (often assumed to be Gaussian noise). Note that here the time lags are negative (“ +*j* ” in the equation above) reflecting the fact that neural activity in the present is being used to predict stimulus values in the past. This is known as an *acausal* relationship because the inputs to the model are not assumed to causally influence the outputs. If the model output corresponds to discrete event types (e.g., different phonemes), then the model is performing *classification*. If the output is a continuously-varying stimulus property such as the power in one frequency band of a spectrogram, the model performs regression and can be used, for example, in *stimulus reconstruction*.

In linear decoding, the weights can operate on a multi-dimensional neural signal, allowing the researcher to consider the joint activity across multiple channels (e.g., electrodes or voxels) around the same time (See **Figure 3**). By fitting a weight to each neural signal, it is possible to infer the stimulus or experiment properties that gave rise to the distributed patterns of neural activity.

The decoder is a proof of concept: given a new pattern of unlabeled brain activity (that is, brain activity *without* its corresponding stimulus properties), it may be possible to reconstruct the most likely stimulus value that resulted in the activity seen in the brain (Naselaris et al., 2009; Pasley et al., 2012). The ability to accurately reconstruct stimulus properties relies on recording signals from the brain that are tuned to a diverse set of stimulus features. If neural signals from multiple channels show a diverse set of tuning properties (and thus if they contain independent information about the stimulus), one may combine the activity of many such channels during decoding in order to increase the accuracy and diversity of decoded stimuli, provided that they carry independent information about the stimulus (Moreno-Bote et al., 2014).

### Benefits of the predictive modeling framework

As discussed above, predictive modeling using multivariate analyses is one of many techniques used in studying the brain. While the relative merits of one analysis over another is not black and white, it is worth discussing specific pros and cons of the framework described in this paper. Below are a few key benefits of the predictive modeling approach.

1. **Generalize on test set data**. Classical statistical tests compare means of measured variables, and statements about significance are based on the error of the point estimates such as the standard error of the mean. When using predictive modeling, cross-validated models are tested for their ability to generalize to new data, and thus are judged against the variability of the population of measurements. As such, classical inferential testing makes statements of statistical significance, while cross-validated encoding/decoding models make statements about the relevance of the model. This allows for more precise statements about the relationship between inputs and outputs. In addition, encoding models offer a continuous measure of model quality, which is a more subtle and complete description of the neural signal being modeled.
2. **Jointly consider many variables**. Many statistical analyses (e.g., Statistical Parametric Mapping fMRI analysis; Friston, 2003) employ massive parallel univariate testing in which variables are first selected if they pass some threshold (e.g., activity in response to auditory stimuli), and subsequent statistical analyses are conducted on this subset of features. This can lead to inflated family-wise error rate and is prone to “double-dipping” if the thresholding is not carried out properly. The predictive modeling approach discussed here uses a multivariate analysis that jointly considers feature values, describing the relative contributions of features as a single weight vector. Because multiple parameters are estimated simultaneously the parameters patterns should be interpreted as a whole. This gives a more complex picture of feature interaction and relative importance, and also reduces the amount of statistical comparisons being made. However, note that it is also possible to perform statistical inference on individual model parameters.
3. **Generate hypotheses with complex stimuli**. Because predictive models can flexibly handle complex inputs and outputs, they can be used as an exploratory step in generating hypotheses about the representation of stimulus features at different regions of the brain. Using the same stimulus and neural activity, researchers can explore hypotheses of stimulus representation at multiple levels of stimulus complexity. This is useful for generating new hypotheses about sensory representation in the brain, which can be confirmed with follow-up experiments.
4. **Discover multivariate structure in the data**. Because predictive models consider input features jointly, they are able to uncover structure in the input features that may not be apparent when testing using univariate methods. For example, STRFs describe complex patterns in spectro-temporal space that are not apparent with univariate testing (see **Figure 5**). It should be noted that any statistical technique will give misleading results if the covariance between features is not taken into consideration, though it is more straightforward to consider feature covariance using the modeling approach described here.
5. **Model subtle time-varying detail in the data**. Traditional statistical approaches tend to collapse data over dimensions such as time (e.g., when calculating a per-trial average). With predictive modeling, it is straightforward to incorporate the relationship between inputs and outputs at each timepoint without treating between-trail variability as noise. This allows one to make statements about the time-varying relationship between inputs and outputs instead of focusing only on whether activity goes up or down on average. Researchers have used this in order to investigate more subtle changes in neural activity such as those driven by subjective perception and internal brain states (Chang et al., 2011; Reichert et al., 2014).

Ultimately, predictive modeling is not a replacement of traditional univariate methods, but should be seen as a complementary tool for asking questions about complex, multivariate inputs and outputs. The following sections describe several types of stimuli and experimental setups that are well-suited for predictive modeling. They cover the general workflow in a predictive modeling framework analysis, as well as a consideration of the differences between regression and classification in the context of encoding and decoding.

## Identifying input/output features

The application of linear regression or classification models requires transforming the stimulus and the neural activity such that they have a linear relationship with one another. This follows the assumption that generally there is a non-linear relationship between measures of neural responses (e.g., spike rate) and those of the raw stimulus (e.g., air pressure fluctuations in the case of speech), but that the relationship becomes *linear* after some non-linear transformation of that raw stimulus (e.g., the speech envelope of the stimulus). The nature of this non-linear transformation is used to investigate what kind of information the neural signal carries about the stimulus. As such, when using the raw stimulus values, a linear model will not be able to accurately model the neural activity, but after a non-linear transformation that matches the transformations performed in the brain, the linear model is now able to explain variance in the neural signal. This is a process called *linearizing* the model (David, 2004; David and Gallant, 2005).

As the underlying math of linear models is straightforward, picking the right set of input/output features is a crucial tool for testing hypotheses. Stimulus linearization can be thought of as a process of *feature extraction/generation*. Features are generally chosen based on previous knowledge or assumptions about a brain region under study, and have been used to investigate the progression of simple to complex feature representations along the sensory pathway.

The following sections describe common feature representations that have been used for building linearized *encoding* and *decoding* models in cognitive electrophysiology. They reflect a restricted set of questions about stimulus transformations in the brain drawn from the literature and are not an exhaustive set of possible questions. Also note that it is possible to use other neural signals as inputs to an encoding model (for example, an autoregressive model uses past timepoints of the signal being predicted as input, which is useful for finding autocorrelations, repeating patterns, and functional connectivity metrics; Bressler and Seth, 2011). However, this article focuses on external stimuli.

### Encoding models

Encoding models define model inputs by decomposing the raw stimulus (be it an image, an audio stream, etc.) into either well-defined high-level features with both a direct relationship with the physical world linked with a particular percept (e.g., spectrogram modulations, center frequencies, cepstral coefficients) or statistical descriptions of these features (e.g., principal or independent components). This is in contrast to a classic approach that builds receptive field maps using spectrograms of white noise used for stimulus generation. The classic approach works well for neural activity in low-level sensory cortex (Marmarelis and Marmarelis, 1978) but results in sub-optimal models for higher-level cortical areas, due in part to the fact that white noise contains no higher-level structure (David, 2004).

**Figure 2:**
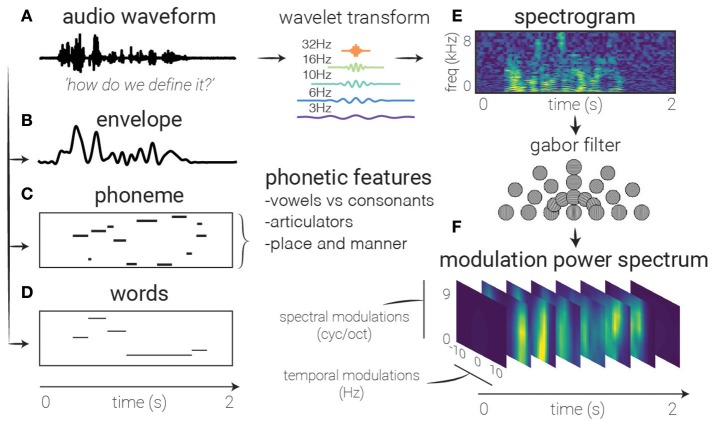
Feature extraction. Several auditory representations are shown for the same natural speech utterance. **(A)** Raw audio. Generally used as a starting point for feature extraction, rarely in linear models, though can be used with non-linear models (and sufficient amounts of data). **(B)** Speech envelope. The raw waveform can be rectified and low-pass filtered to extract the speech envelope, representing the amount of time-varying energy present in the speech utterance. **(E)** Spectrogram. A time-frequency decomposition of the raw auditory waveform can be used to generate a spectrogram that reflects spectro-temporal fluctuations over time, revealing spectro-temporal structure related to higher-level speech features. **(F)** Modulation Power Spectrum. A two-dimensional Gabor decomposition of the spectrogram itself can be used to create the MPS of the stimulus, which summarizes the presence or absence (i.e., power) of specific spectro-temporal fluctuations in the spectrogram. **(C)** Phonemes. In contrast to previous features which are defined acoustically, one may also use linguistic features to code the auditory stimulus, in this case with categorical variables corresponding to the presence of phonemes. **(D)** Words. Another higher-order feature that is not directly related to any one spectrotemporal pattern, these types of features may be used to investigate higher-level activity in the brain's response.

The study of sound coding in early auditory cortices commonly employs a windowed decomposition of the raw audio waveform to generate a spectrogram of sound—a description of the spectral content in the signal as it changes over time (see Figure 2). Using a spectrogram as input to a linear model has been used to create a *spectro-temporal receptive field* STRF. This can be interpreted as a filter that describes the spectro-temporal properties of sound that elicit an increase in activity in the neural signal. The STRF is a feature representation used to study both single unit behavior (Aertsen and Johannesma, 1981; Theunissen et al., 2000; Depireux et al., 2001; Sen et al., 2001; Escabí and Schreiner, 2002) and human electrophysiology signals (Pasley and Knight, 2012; Di Liberto et al., 2015; Holdgraf et al., 2016; Hullett et al., 2016).

It should be noted that spectrograms (or other time-frequency decompositions) are not the only way to represent auditory stimuli. Others researchers have used cepstral decompositions of the spectrogram (Hermansky and Morgan, 1994), which embed perceptual models within the definition of the stimuli features or have chosen stimulus feature representations that are thought to mimic the coding of sounds in the sensory periphery (Chi et al., 2005; Pasley et al., 2012). Just as sensory systems are believed to extract features of increasing abstraction as they continue up the sensory processing chain, researchers have used features of increasing complexity to model higher-order cortex (Sharpee et al., 2011). For example, while spectrograms are used to model early auditory cortices, researchers often perform a secondary non-linear decomposition on the spectrograms to implement hypothesized transformations implemented in the auditory hierarchy such as phonemic, lexical, or semantic information. These are examples of *linearizing* the relationship between brain activity and the stimulus representation.

In one approach, the energy modulations across both time and frequency are extracted from a speech spectrogram by using a filter bank of two-dimensional Gabor functions (see Sidenote on Gabors). This results extracts the Modulation Power Spectrum of the stimulus (in the context of receptive fields, also called the Modulation Transfer Function). This feature representation has been used to study higher-level regions in auditory cortex (Theunissen et al., 2001; Chi et al., 2005; Elliott and Theunissen, 2009; Pasley et al., 2012; Santoro et al., 2014). There have also been efforts to model brain activity using higher-order features that are not easily connected to low-level sensory features, such as semantic categories (Huth et al., 2016). This also opens opportunities for studying more abstract neural features such as the activity of a distributed network of neural signals.

Alternatively, one could create features that exploit the stimulus statistics, for example features that are made statistically independent from each other (Bell and Sejnowski, 1995) or by exploiting the concept of sparsity of stimulus representation bases (Olshausen and Field, 1997, 2004; Shelton et al., 2015). Feature sparseness of can improve the predictive power and interpretability of models because the representation of stimulus features in active neural populations may be inherently sparse (Olshausen and Field, 2004). For example, researchers have used the concept of sparseness to learn model features from the stimuli set by means of an unsupervised approach that estimates the primitives related to the original stimuli (e.g., for vision: configurations of 2-D bars with different orientations). This approach is also known as “dictionary learning” and has been used to model the neural response to simple input features in neuroimaging data (Henniges and Puertas, 2010; Güçlü and van Gerven, 2014). It should be noted that more “data-driven” methods for feature extraction often discover features that are similar to those defined *a priori* by researchers. For example, Gabor functions have proven to be a useful way to describe both auditory (Lewicki, 2002) and visual (Touryan et al., 2005) structure, and are both commonly used in the neural modeling literature. In parallel, methods that attempt to define features using methods that maximize between-feature statistical independence (such as Independent Components Analysis) also often discover features that look similar to Gabor wavelets (Olshausen and Field, 1997; see *Sidenote on Gabors* for more detail^2^).

It is also possible to select different neural *output* features (e.g., power in a particular frequency band of the LFP) to ask different questions about neural activity. The choice of neural feature impacts the model's ability to predict patterns of activity, as well as the conclusions one may draw from interpreting the model's weights. For example, encoding models in electrocorticography are particularly useful because of “high-frequency” activity (70–200 Hz) that reflects local neural processing (Ray and Maunsell, 2011). This signal has a high signal-to-noise ratio, making it possible to fit models with more complicated features. Since it is tightly linked to ensembles of neurons, it is more straightforward to interpret how the stimulus features are encoded in the brain (Pasley et al., 2012; Hullett et al., 2016) and to connect with the single-unit encoding literature (Theunissen and Elie, 2014). Researchers have also used more complex representations of neural activity to investigate the type of information they may encode. For example, in order to investigate the interaction between attention and multiple speech streams (Zion Golumbic et al., 2013), computed a “temporal receptive field” of an auditory speech envelope for theta activity in ECoG subjects. A similar analysis has been performed with EEG (Di Liberto et al., 2015). It is also possible to describe patterns of distributed activity in neural signals (e.g., using Principle Components Analysis or network activity levels), and use this as the output being predicted [though this document treats each output (i.e., channel) as a single recording unit].

An important development in the field of linear encoding models is loosening of the assumptions of stationarity to treat the input/output relationship as a dynamic process (Meyer et al., 2017). While a single model assumes stationarity in this relationship, fitting multiple models on different points in time or different experimental conditions allows the researcher to make inferences about how (and why) the relationship between stimulus features and neural activity changes. For example, Fritz et al. recorded activity in the primary auditory cortex of ferrets during a tone frequency detection task (Fritz et al., 2005). The authors showed that STRFs of neurons changed their tuning when the animal was actively attending to a frequency vs. passively listening to stimuli, suggesting that receptive fields are more plastic than classically assumed (Meyer et al., 2014). Further support for dynamic encoding is provided by Holdgraf et al. who implemented a task in which ECoG subjects listened to degraded speech sentences. A degraded speech sentence was played, followed by an “auditory context” sentence, and then the degraded speech was repeated. The context created a powerful behavioral “pop-out” effect whereby the degraded speech was rendered intelligible. The authors compared the STRF of electrodes in the auditory cortex in response to degraded speech *before* and *after* this context was given, and showed that it exhibited plasticity that was related to the perceptual “pop-out” effect (Holdgraf et al., 2016). Our understanding of the dynamic representation of low-level stimulus features continues to evolve as we learn more about the underlying computations being performed by sensory systems, and the kinds of feature representations needed to perform these computations (Thorson et al., 2015).

### Decoding models

While decoding models typically utilize the same features as encoding models, there are special precautions to consider because inputs and outputs are reversed relative to encoding models. Speech decoding is a complex problem that can be approached with different goals, strategies, and methods. In particular, two main categories of decoding models have been employed: classification and reconstruction.

In a classification framework, the neural activity during specific events is identified as belonging to one of a finite set of possible event types. For instance, one of six words or phrases. There are many algorithms (linear and non-linear) for fitting a classification model, such as support-vector machines, Bayesian classifiers, and logistic regression (Hastie et al., 2009). All these algorithms involve weighting input features (neural signals) and outputting a discrete value (the class of a datapoint) or a value between 0 and 1 (probability estimate for the class of a datapoint). This may be used to predict many types of discrete outputs, such as the trial or stimulus “types” (e.g., consonant vs. dissonant chords), image recognition (Rieger et al., 2008), finger movements (Quandt et al., 2012), social decisions (Hollmann et al., 2011), or even subjective conscious percepts (Reichert et al., 2014). In this case, the experimental design requires a finite number of repetitions of each stimulus type (or class). In speech research, discrete speech features have been predicted above chance levels, such as vowels and consonants (Pei et al., 2011; Bouchard and Chang, 2014), phonemes (Chang et al., 2010; Brumberg et al., 2011; Mugler et al., 2014), syllables (Blakely et al., 2008), words (Kellis et al., 2010; Martin et al., 2016), sentences (Zhang et al., 2012), segmental features (Lotte et al., 2015), and semantic information (Degenhart et al., 2011).

In a reconstruction approach, continuous features of the stimulus are reconstructed to match the original feature set. For instance, upper limb movement parameters, such as position, velocity, and force were successively decoded to operate a robotic arm (Hochberg et al., 2012). In speech reconstruction, features of the sound spectrum, such as formant frequencies (Brumberg et al., 2010), amplitude power, and spectrotemporal modulations (Pasley et al., 2012; Martin et al., 2014, 2016), mel-frequency cepstral-coefficients (Chakrabarti et al., 2013), or the speech envelope (Kubanek et al., 2013) have been accurately reconstructed. In a recent study, formant frequencies of intended speech were decoded in real-time directly from the activity of neurons recorded from intracortical electrodes implanted in the motor cortex, and speech sounds were synthesized from the decoded acoustic features (Brumberg et al., 2010).

While both encoding and decoding models are used to relate stimulus features and neural activity, decoding models have an added potential to be used in applications that attempt to use patterns of neural activity to control physical objects (such as robotic arms) or predict the stimulus properties underlying the neural activity (such as inner speech prediction). These are both examples of neural prosthetics, which are designed to utilize brain activity to help disabled individuals interact with the world and improve their quality of life. However, it is also possible (and preferable in some cases) to decode stimulus properties *using an encoding model*. In this case, encoding model parameters may be used to build probability distributions over the most likely stimulus properties that resulted in a (novel) pattern of brain activity (Kay et al., 2008; Naselaris et al., 2011; Nishimoto et al., 2011).

In summary, linearizing stimulus features allows one to use linear models to find non-linear relationships between datasets. This approach is simpler, requires less computation, and is generally more interpretable than using non-linear models, and is flexible with respect to the kinds of features chosen (Naselaris et al., 2011; Shamma, 2013; de Heer et al., 2017). The challenge often lies in choosing these features based on previous literature and the hypothesis one wants to test, and interpreting the resulting model weights (see Interpreting the Models section, as well as Figure 2 for a description of many features used in predictive modeling).

## Choosing and fitting the model

**Figure 3:**
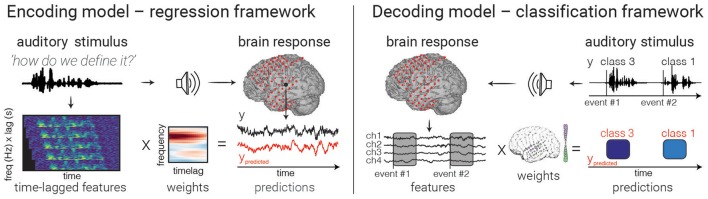
Model fitting. An example of encoding **(left)** and decoding **(right)** models are depicted. In encoding models, one attempts to predict the neural activity from the auditory representation by finding a set of weights (one for each feature/time lag) that minimizes the difference between true (black) and predicted (red) values. In decoding with a classifier (right), brain activity in multiple electrodes is used to make a discrete prediction of the category of a stimulus. Note that decoding models can also use regression to predict continuous auditory feature values from brain activity, though only classification is shown above.

After choosing stimulus features (as inputs to an encoding model, or outputs to a decoding model) as well as the neural signal of interest, one must link these two data sets by “fitting” the model. The choice of modeling framework will influence the nature of the inputs and outputs, as well as the questions one may ask with it. This section discusses common modeling frameworks for encoding and decoding (see Figure 3 for a general description of the components that make up each modeling framework). It focuses on the linear model, an approach that has proven to be powerful in answering complex questions about the brain. We highlight some caveats and best-practices.

### Choosing a modeling framework

The choice of modeling framework affects the relationship one may find between inputs and outputs. Finding more complex relationships usually requires more data and is prone to overfitting, while finding simpler relationships can be more straightforward and efficient, but runs the risk of missing a more complex relationship between inputs and outputs.

While many model architectures have been used in neural modeling, this paper focuses on those that find linear relationships between inputs and outputs. We focus on this case because of the ubiquity and flexibility of linear models, though it should be noted that many other model structures have been used in the literature. For example, it is common to include non-linearities on the *output* of a linear model (e.g., a sigmoid that acts as a non-linear suppression of output amplitude). This can be used to transform the output into a value that corresponds to neural activity such as a Poisson firing rate (Paninski, 2004; Christianson et al., 2008), to incorporate knowledge of the biophysical properties of the nervous system (McFarland et al., 2013), to incorporate the outputs of other models such as neighboring neural activity (Pillow et al., 2011), or to accommodate a subsequent statistical technique (e.g., in logarithmic classification, see above). It is also possible to use summary statistics or mathematical descriptions of the receptive fields described above as inputs to a subsequent model (Thorson et al., 2015).

It is possible to fit non-linear models directly in order to find more complex relationships between inputs and outputs. These may be an extension of linear modeling, such as models that estimate input non-linearities (Ahrens et al., 2008), spike-triggered covariance (Paninski, 2003; Schwartz et al., 2006), and other techniques that fit multi-component linear filters for a single neural output (Sharpee et al., 2004; Meyer et al., 2017). Note that, after projecting the stimulus into the subspace spanned by these multiple filters, the relationship between this projection and the response can be non-linear, and this approach can be used to estimate the higher-order terms of the stimulus-response function (Eggermont, 1993). While non-linear methods find a more complicated relationship between inputs and outputs, they may be hard to interpret (but see Sharpee, 2016), require significantly more data in order to generalize to test-set data, and often contain many more free-parameters that must be tweaked to optimize the model fit (Ahrens et al., 2008). In addition, optimization-based methods for fitting these models generally requires traversing a more complex error landscape, with multiple local minima that do not guarantee that the model will converge upon a global minimum (Hastie et al., 2009).

As described in section Identifying Input/Output Features, generalized linear models provide the complexity of non-linear feature transformations (in the form of feature extraction steps) with the simplicity and tractability of a linear model. For this reason linear modeling has a strong presence in neuroscience literature, and will be the focus of this manuscript. See (Meyer et al., 2017) for an in-depth review of many (linear and non-linear) modeling frameworks that have been used in neural encoding and decoding.

### The least-squares solution

As described above, generalized linear models offer a balance between model complexity and model interpretability. While any kind of non-linear transformation can be made to raw input or output features *prior* to fitting, the model itself will then find *linear* relationships between the input and output features. At its core, this means finding one weight per feature such that, when each feature is weighted and summed, it either minimizes or maximizes the value of some function (often called a “cost” function). A common formulation for the cost function is to include “loss” penalties such as model squared error (Hastie et al., 2009) on both the training and the validation set of data. The following paragraphs describe a common way to define the loss (or error) in linear regression models, and how this can be used to find values for model coefficients.

In the case of least-squares regression, we define the predictions of a model as the dot product between the weight vector and the input matrix:

$$\begin{array}{l} \hat{y}=Xw \end{array}$$

In this case, the cost function is simply the squared difference between the predicted values and the actual values for the output variable. It takes the following form:

$$\begin{array}{l} CF_{LS}=error=\frac{1}{n}{\left(\hat{y}-y\right)}^{T}\left(\hat{y}-y\right) \end{array}$$

In this case, ***X*** is the input training data and ***w*** are the model weights, and the term $\hat{y}$ represents model predictions given a set of data. *y* is the “true” output values, and *n* is the total number of data points. Both ***y*** and $\hat{y}$ are column vectors where each row is a point in time. *CF*_*LS*_ stands for the “least squares” cost function. In this case it contains a single loss function that measures the average squared difference between model predictions and “true” outputs.

If there are many more data points than features (a rule of thumb is to have at least 10 times more data points than features, though this is context-dependent), then finding a set of weights that minimizes this loss function (the squared error) has a relatively simple solution, known as the *Least Squares Solution* or the *Normal equation*. It is the solution obtained by maximum likelihood with the assumption of Gaussian error. The least square solution is:

$$\begin{array}{l} weights_{LS}={\left(X^{T}X\right)}^{-1}X^{T}y\; \end{array}$$

Where ***X*** is the (*n* time points or observations by *m* features) input matrix, and *y* is an output vector of length *n observations*. When ***X*** and ***y*** have a mean of zero, the expression ($\frac{{\text{X}}^{\text{T}}\text{y}}{\text{n}}$) is the cross-covariance between each input feature and the output. This is then normalized by the auto-covariance matrix of the input features ($\frac{{\text{X}}^{\text{T}}\text{X}}{\text{n}}$). The output will be a vector of length *m* feature weights that defines how to mix input features together to make one predicted output. It should be noted that while this model weight solution is straightforward to interpret and quick to find, it has several drawbacks such as a tendency to “overfit” to data, as well as the inability to impose relationships between features (such as a smoothness constraint). Some of these will be discussed further in section From Regression to Classification, Using Regularization to Avoid Overfitting.

### From regression to classification

While classification and regression seem to perform very different tasks, the underlying math between them is surprisingly similar. In fact, a small modification to the regression equations results in a model that makes predictions between two classes instead of outputting a continuous variable. This occurs by taking the output of the linear model and passing it through a function that maps this output onto a number representing the probability that a sample comes from a given class. The function that does this is called the *link function*.

$$\begin{array}{l} p_{class}=f^{-1}\left(Xw+b\right) \end{array}$$

Where *p* is the probability of belonging to one of the two classes and *f*^−1^ is the inverse of the link function (called the *inverse link function*). For example, in logistic regression, *f* is given by the logistic function:

$$\begin{array}{l} \log\left(\frac{p}{1-p}\right)=\;Xw+b \end{array}$$

***Xw*** is the weighted sum of the inputs, and the scalar **(*b*)** is a bias term. Taken together, this term defines the angle **(*w*)** and distance from origin **(*b*)** of a line in feature space that separates the two classes, often called the *decision plane*.

Datapoints will be categorized as belonging to one class of another depending on which side of the line they lie. The quantity ***X**w* + *b* provides a normalized distance from each sample in ***X*** to the classifier's decision plane (which is positioned at a distance, ***b***, from the origin). This distance can be associated with a particular probability that the sample belongs to a class. Note that one can also use a step function for the link function, thus generating binary YES/NO predictions about class identity.

While the math behind various classifiers will differ, they are all essentially performing the same task: define a means of “slicing up” feature space such that datapoints in one or another region of this space are categorized according to that region's respective class. For example, *Support Vector Machines* also find a linear relationship that separates classes in feature spaces, with an extra constraint that controls the distance between the separating line and the nearest member of each class (Hastie et al., 2009).

### Using regularization to avoid overfitting

The analytical least-squares solution is simple, but often fails due to *overfitting* when there are a high number of feature dimensions (*m*) relative to observations (*n*). In overfitting, the weights become too sensitive to fluctuations in the data that would average to zero in larger data sets. As the number of parameters in the model grows, this sensitivity to noise increases. Overfitting is most easily detected when the model performs well on the training data, but performs poorly on the testing data (see section Validating the Model).

Neural recordings are often highly variable either because of signal to noise limitations of the measures or because of the additional difficulty of producing a stationary internal brain state (Theunissen et al., 2001; Sahani and Linden, 2003). At the same time, there is increasing interest in using more complex features to model brain activity. Moreover, the amount of available data is often severely restricted, and in extreme cases there are fewer datapoints than weights to fit. In these cases the problem is said to be *underconstrained*, reflecting the fact that there is not enough data to properly constrain the weights of the model. To handle such situations and to avoid overfitting the data, it is common to employ *regularization* when fitting models. The basic goal of regularization is to add constraints (or equivalently priors) on the weights to effectively reduce the number of parameters (*m*) in the model and prevent overfitting. Regularization is also called shrinking, as it shrinks the number or magnitude of parameters. A common way to do this is to use a penalty on the total magnitude of all weight values. This is called imposing a “norm” on the weights. In the Bayesian framework, different types of penalties correspond to different priors on the weights. They reflect assumptions on the probability distribution of the weights *before* observing the data (Wu et al., 2006; Naselaris et al., 2011).

In machine learning, norms follow the convention l*N*, where *N* is generally 1 or 2 (though it could be any value in between). Constraining the norm of the weights adds an extra term to the model's cost function, combining the traditional least squares loss function with a function of the magnitude across all weights. For example, using the 12 norm (in a technique called *Ridge Regression*) adds an extra penalty to the squared sum of all weights, resulting in the following value for the regression cost function:

$$\begin{array}{l} CF_{Ridge}=\frac{1}{n}{\left(Xw-y\right)}^{2}+\lambda {\left||w|\right|}^{2} \end{array}$$

Where *w* is the model weights, *n* is the number of samples, and λ is a hyper-parameter (in this case called the Ridge parameter) that controls the relative influence between the weight magnitude vs. the mean squared error. Ridge regression corresponds to a Gaussian prior on the weight distribution with variance given by $\frac{1}{\lambda }.$ For small values of λ, the optimal model fit will be largely driven by the squared error, for large values, the model fit will be driven by minimizing the magnitude of model weights. As a result, all of the weights will trend toward smaller numbers. For Ridge regression, the weights can also be obtained analytically:

$$\begin{array}{l} weights_{Ridge}={\left(X^{T}X+I\lambda \right)}^{-1}X^{T}y\; \end{array}$$

There are many other forms of regularization, for example, ℓ1 regularization (also known as Lasso Regression) adds a penalty for the sum of the absolute value of all weights and causes many weights to be close to 0, while a few may remain larger (known as fitting a *sparse* set of weights). It is also common to simultaneously balance ℓ1 and ℓ2 penalties in the same model (called *Elastic* Nets, (Hastie et al., 2009)).

In general, regularization tends to reduce the *variance* of the weights, restricting them to a smaller space of possible values and reducing their sensitivity to noise. In the case of *lN* regression, this is often described as placing a finite amount of magnitude that is spread out between the weights. The *N* in ℓ*N* regression controls the extent to which this magnitude is given to a small subset of weights vs. shared equally between all weights. For example, in Ridge regression, large weights are penalized more, which encourages all weights to be smaller in value. This encourages weights that smoothly vary from one to another, and may discourage excessively high weights on any one weight which may be due to noise. Regularization reduces the likelihood that weights will be overfit to noise in the data and improves the testing data score. ℓ2 regularization also has the advantage of having an analytical solution, which can speed up computation time. An exhaustive description of useful regularization methods and their effect on analyses can be found in Hastie et al. (2009).

Parameters that are not directly fit to the training data (such as the Ridge parameter) are called *hyper-parameters* or *free parameters*. They exist at a higher level than the fitted model weights, and influence the behavior of the model fitting process in different ways (e.g., the number of non-zero weights in the model, or the extent to which more complex model features can be created out of combinations of the original features). They are not determined in the standard model fitting process, however they can be chosen in order to minimize the error on a validation dataset (see below). Changing a hyper-parameter in order to maximize statistics such as prediction score is called *tuning* the parameter, which will be covered in the next section.

In addition, there are many choices made in predictive modeling that are not easily quantifiable. For example, the choice of the model form (e.g., ℓ2 vs. ℓ1 regularization) is an additional free model parameter that will affect the result. In addition, there are often multiple ways to “fit” a model. For example, the least-squares solution is not always solved in its analytic form. If the number of features is prohibitively large, it is common to use numerical approximations to the above equation, such as gradient descent, which uses an iterative approach to find the set of weights that minimizes the cost function. With linear models that utilize enough independent data points, there is always one set of weight parameters that has the lowest error, often described as a “global minimum.” In contrast, non-linear models have a landscape of both local and global minima, in which small changes to parameter values will *increase* model error and so the gradient descent algorithm will (incorrectly) stop early. In this way, iterative methods may get “stuck” in a local minimum without reaching a global minimum. Linear models do not suffer from the problem of local minima. However, since gradient descent often stops before total convergence, it may result in (small) variations in the final solution given different weight initializations.

Note that for linear time-invariant models (i.e., when the weights of the model do not change over time) and when the second order statistical properties of the stimulus are stationary in time (i.e., the variance and covariance of the stimulus do not change with time), then it is more efficient to find the linear coefficients of the model in the Fourier domain. For stimuli with those time-invariant properties, the eigenvectors of the stimulus auto-covariance matrix ($\frac{X^{T}X}{n}$ in the normal equation) are the discrete Fourier Transform. Thus, by transforming the cross-correlation between the stimulus and the response ($\frac{X^{T}y}{n}$) into the frequency domain, the normal equation becomes a division of the Fourier representation of *X*^*T*^*y* and the power of the stimulus at each frequency. Moreover, by limiting the estimation of the linear filter weights to the frequencies with significant power (i.e., those for which there is sufficient sampling in the data), one effectively regularizes the regression. See (Theunissen et al., 2001) for an in-depth discussion.

## Validating the model

After data have been collected, model features have been determined, and model weights have been fit, it is important to determine whether the model is a “good” description of the relationship between stimulus features and brain activity. This is called *validating* the model. This critical step involves making model predictions using new data and determining if the predictions capture variability in the “ground truth” of data that was recorded.

Validating a model should be performed on data that was not used to train the model, including preprocessing, feature selection, and model fitting. It is common to use *cross-validation* to accomplish this. In this approach, the researcher splits the data into two subsets. One subset is used to train the model (a “training set”), and the other is used to validate the model (a “test set”). If the model has captured a “true” underlying relationship between inputs and outputs, then the model should be able to accurately predict data points that it has never seen before (those in the test set). This gives an indication for the stability of the model's predictive power (e.g., how well is it able to predict different subsets of held-out data), as well as the stability of the model weights (e.g., placing confidence intervals on the weight values).

**Figure 4:**
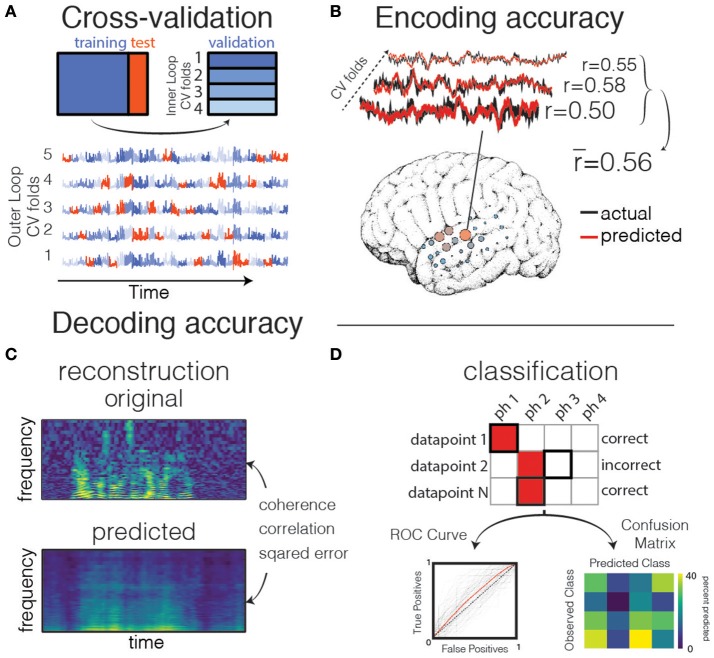
Validation and prediction. **(A)** Cross validation is used to tune hyperparameters and validate the model. In one iteration of the outer loop, the data is split into training and test sets. Right: an inner loop is then performed on the training set, with a different subset of training data (blue shades) held out as a validation set for assessing hyperparameter performance. The hyperparameter with the highest mean score across inner-loop iterations is generally chosen for a final evaluation on the test set. Lower: The same neural timeseries across five iterations of the outer-loop. Each iteration results in a different partitioning of the data into training, test, and validation sets. Note that timepoints are grouped together in time to avoid overfitting during hyperparameter tuning. **(B)** Examples of actual and predicted brain activity for various cross-validated testing folds. The overall prediction score is averaged across folds, and displayed on the surface of the subject's reconstructed brain. **(C)** In decoding models when performing stimulus reconstruction (regression), a model is fit for each frequency band. Model predictions may be combined to create a predicted spectrogram. The predicted and original auditory spectrograms are compared using metrics such as mean squared error. **(D)** When using classification for decoding, the model predicts one of several classes for each test datapoint. These predictions are validated with metrics such as the Receiver Operating Characteristic (ROC, left) that shows the performance of a binary classifier system as its discrimination threshold is varied. The ROC curve is shown for each outer CV iteration (black) as well as the mean across CV iterations (red). If the classifier outputs the labels above chance level, the Area Under the ROC curve (AUC) will be larger than 0.5. Alternatively, the model performance can be compared across classes resulting in a confusion matrix (right), which shows for what percent of the testing set a class was predicted (columns) given the actual class (rows). The ith row and jth column represents the percent of trials that a datapoint of class *i* was predicted to belong to class *j*.

There are many ways to perform cross-validation. For example, in *K*-fold cross validation, the dataset is split into *K* subsets (usually between 5 and 10). The model is fit on *K-1* subsets, and then validated on the held-out subset. The cross validation iterates over these sets until each subset was once a test set. In the extreme case, there are as many subsets as there are datapoints, and a single datapoint is left out for the validation set on each iteration. This is called Leave One Out cross validation, though it may bias the results and should only be used if very little data for training the model is available (Varoquaux et al., 2016). Because electrophysiology data is correlated with itself (i.e., autocorrelated) in time, it is crucial when creating training/test splits to avoid separating datapoints that occur close to one another in time (for example, by keeping “chunks” of contiguous timepoints together, such as a single trial that consists of one spoken sentence). If this is not done, correlations between datapoints that occur close to one another in time will artificially inflate the model performance when they occur in both the training and test sets. This is because the model will be effectively trained and tested on the same set of data, due to patterns in both the signal and the noise being split between training/test sets. See Figure 4 for a description of the cross-validation process, as well as the Jupyter notebook “Prediction and Validation,” section “Aside: what happens if we don't split by trials?”^3^

Determining the correct hyper-parameter for regularization requires an extra step in the cross-validation process. The first step is the same: the full dataset is split into two parts, training data and testing data (called the “outer loop”). Next, the training data is split once more into training and validation datasets (called the “inner loop”). In the inner loop, a range of hyper-parameter values is used to fit models on a subset of the training data, and each model is validated on the held-out validation data, resulting in one model score per hyper-parameter value for each iteration of the inner loop. The “best” hyper-parameter is chosen by aggregating across inner loop iterations, and choosing the hyper-parameter value with the best model performance. The model with this parameter is then re-tested on the outer loop testing data. The process of searching over many possible hyper-parameter values is called a “grid search,” and the whole process of splitting training data into subsets of training/validation data is often called nested-loop cross validation. Efficient hyper-parameter search strategies exist for some learning algorithms (Hastie et al., 2009). However, there are caveats to doing this effectively, and the result may still be biased with particularly noisy data (Varoquaux et al., 2016).

### Metrics for regression prediction scores

As described previously, inputs and outputs to a predictive model are generally created using one or more non-linear transformations of the raw stimulus and neural activity. The flexible nature of inputs and outputs in regression means that there are many alternative fitted models. In general, a model's performance is gauged from its ability to make predictions about data it has never seen before (data in a validation or test set) requiring a criterion to perform objective comparisons among all those models. The definition of model performance depends on the type of output for the model (e.g., a time series in regression vs. a label in categorization). It will also depend on the metric of error (or loss function) used, which itself depends on assumptions about the noise inherent in the system (e.g., whether it is normally-distributed). Assumptions about noise will depend on both the neural system being studied (e.g., single units vs. continuous variables such as high-frequency activity in ECoG) as well as the kind of model being used (Paninski, 2004). The metric of squared error (described below) assumes normally-distributed noise, and will be assumed for continuous signals in the remainder of the text.

#### Coefficient of determination (*R*^2^)

Encoding models as well as decoding models for stimulus reconstruction use regression, which outputs a continuously varying value. The extent to which regression predictions match the actual recorded data is called model *goodness of fit (GoF)*. A robust measure is the *Coefficient of Determination (R*^2^*)*, defined as the squared error between the predicted and actual activity, divided by the squared error that would have occurred with a model that simply predicts the mean of the true output data.

$$\begin{array}{l} SSE_{tot}=\sum_{i}{\left(y_{i}-\bar{y}\right)}^{2}\\ SSE_{reg}=\sum_{i}{\left(y_{i}-\;{\hat{y}}_{i}\right)}^{2}\\ R^{2}=1-\frac{SSE_{reg}}{SSE_{tot}} \end{array}$$

where ŷ_*i*_ is the predicted value of *y* at timepoint *i*, and ȳ is the mean value of *y* over all timepoints. The first two terms are both called the *sum of squared error. One* is the error defined by the model (the difference between predicted and actual values), and the other is the error defined by the output's deviation around its own mean (closely related to the output variance). Computing the ratio of errors provides an index for the increase in output variability explained by the regression model. If *R*^2^ is positive it means that the variance of the model's error is less than the variance of the testing data, if it is zero then the model makes predictions no better than a model that simply predicts the mean of the testing data, and if it is negative then the variance of the model's error is larger than the variance of the testing data (this is only possible when the linear model is being tested on data on which it was not fit).

The Coefficient of Determination, when used with a linear model and without cross-validation, is related to Pearson's correlation coefficient, *r*, by *R*^2^ = *r*^2^. However, on held-out data *R*^2^ can be negative whereas the correlation coefficient squared (*r*^2^) must be positive. Finally, *R*^2^ is directly obtained from the sum of square errors which is the value that is minimized in regression with normally-distributed noise. Thus, it is a natural choice for GoF in the selection of the best hyper-parameter in regularized regression.

#### Coherence and mutual information

Another option for assessing model performance in regression is coherence. This approach uses Fourier methods to assess the extent to which predicted and actual signals share temporal structure. This is a more appropriate metric when the predicted signals are time series, and is given by the following form:

$$\begin{array}{l} \gamma {\left(\omega \right)}^{2}=\frac{\langle X\left(\omega \right)Y^{*}\left(\omega \right)\rangle \langle X^{*}\left(\omega \right)Y\left(\omega \right)\rangle }{\langle X\left(\omega \right)X^{*}\left(\omega \right)\rangle \langle Y^{*}\left(\omega \right)Y\left(\omega \right)\rangle } \end{array}$$

where *X*(ω) and *Y*(ω) are complex numbers representing the stimulus and neural Fourier component at frequency ω, and X^*^(ω) represents the complex conjugate. It is common to calculate the coherence at each frequency, ω, and then convert the output into *Gaussian Mutual Information (MI)*, an information theoretic quantity with units of *bits*/*sec* (also known as the channel capacity) that characterizes an upper bound for information transmission for signals with a particular frequency power spectrum, and for noise with normal distributions. The Gaussian MI is given by:

$$\begin{array}{l} MI_{norm}\left(\omega \right)=-\overset{\infty }{\underset{0}{\int }}log2\left(1-\gamma^{2}\left(\omega \right)\right)d\omega \end{array}$$

While this metric is more complex than using *R*^2^, it is well-suited to the temporal properties of neural timeseries data. In particular, it provides a data-driven approach to determining the relevant time scales (or bandwidth) of the signal and circumvents the need for smoothing the signal or its prediction before estimating GoF values such as *R*^2^ (Theunissen et al., 2001).

### Metrics for classification prediction scores

#### Common statistics and estimating baseline scores

It is common to use *classification* models in decoding, which output a discrete variable in the form of a predicted class identity (such as a brain state or experimental condition). In this case, there is a simple “yes/no” answer for whether the prediction was correct. As such, it is common to report the percent correct of each class type for model scoring. This is then compared to a percent correct one would expect using random guessing (e.g., $100*\frac{1}{n_{classes}}$). If there are different numbers of datapoints represented in each class, then a better baseline is the percentage of datapoints that belong to the most common class (e.g., $100*\frac{n_{A}}{n_{A}+\;n_{B}}$). It should be noted that these are *theoretical* measures of guessing levels, but a better guessing level can often be estimated from the data (Rieger et al., 2008). For example, it is common to use a permutation approach to randomly distribute labels among examples in the training set, and to repeat the cross validation several hundred times to obtain an estimate of the classification rate that can be obtained with such “random” datasets. This classification rate then serves as the “null” baseline. This approach may also reveal an unexpected transfer of information between training and test data that leads to an unexpectedly high guessing level.

#### ROC curves

It is often informative to investigate the behavior of a classifier when the bias parameter, ***b***, is varied. Varying ***b*** and calculating the ratio of “true-positive” to “false-positives” creates the *Receiver Operating Characteristic* (ROC) curve of the classifier (Green and Swets, 1988). This describes the extent to which a classifier is able to separate the two classes. The integral over the ROC curve reflects the separability of the two classes independent of the decision criterion, providing a less-biased metric than percent correct (Hastie et al., 2009).

A geometric interpretation may help to understand how the ROC curve is calculated. The classifier's decision surface is an oriented plane in the space spanned by the features (e.g., a line in a 2-D space, if there are only two features). In order to determine the class of each sample, the samples are projected onto the normal vector of the decision plane by calculating ***Xw***. Samples on one side of the place will result in a positive value for ***Xw***, while samples on the other side of the plane will be negative. This corresponds to the two classes, and results in two histograms for the values of ***Xw***, one for each class. The decision criterion *informativetoinvestigate* can then be varied, resulting in different separations of the samples into two classes. By varying ***b*** for a range of values, and comparing the *predicted* vs. the *true* labels for each value of ***b***, one calculates false positives (false alarms) and true positives (hits) for several decision planes with the same orientation but different positions. Calculating these values for many positions of the decision boundary constructs the ROC curve. A demonstration of the ROC curve and how it relates to the model's hyperplane can be found in the provided jupyter notebooks.

The Area Under the Curve (AUC) is simply the total amount of area under the ROC curve, and is often reported as a summary statistic of the ROC curve. If the classifier is performing at chance, then the AUC will be 0.5, and if it correctly labels all datapoints for all decision thresholds, then the AUC will be 1. More advanced topics relating to classifier algorithms are covered in Hastie et al. (2009) and Pedregosa et al. (2011).

#### The confusion matrix

In the case of multi-class classification (e.g., multinomial logistic regression), it is common to represent the results using a *confusion matrix*. In this visualization, each row is the “known” class, and each column is a predicted class. The *i, j*th value represents the number of times that a datapoint known to belong to class *i* was predicted to belong to class *j*. As such, the diagonal line represents correct predictions (where *class*_*true*_ = *class*_*predicted*_), and any off-diagonal values represent incorrect predictions (see Figure 4D).

Confusion matrices are useful because they describe a more complex picture of how the model predictions perform. This makes it possible to account for more complex patterns in the model's predictions. To capture information about systematic errors (for example if stimulus labels fall into subsets of groups between which the model cannot distinguish), one can use confusion matrices to estimate the mutual information that fully describes the joint probabilities between the predicted class and the actual class (e.g., Chang et al., 2010; Elie and Theunissen, 2016).

### What is a “good” model score?

Determining whether a model's predictive score is “good” or not is not trivial. Many regression and classification scoring metrics are a continuously varying number, and deciding a cutoff point above which a score is not only “statistically significant” but also large enough in effect size to warrant reporting is a challenging problem. This is particularly critical for applications such as Brain Computer Interfaces.

#### Statistical significance

A common practice in model fitting is to determine which models pass some criteria for statistical “significance.” This usually means assessing whether the model is able to make predictions above chance (e.g., a coefficient of determination significantly different from zero in the case of regression, or an AUC > 0.5 in the case of classification). To assess importance and model generalizability, the researcher needs to compare the prediction of the new model to those obtained in other models (i.e., with other feature spaces or other, usually simpler, architectures). If improvements in GoF are clearly observed, then the researcher may investigate the model properties (such as the model weights) to determine which features were most influential in predicting outputs.

As mentioned above, there are multiple challenges with using predictive power to assess the performance of an encoding/decoding model. When fitting model parameters, most models assume that output signals have independent and either Gaussian- or Poisson-distributed noise. If this assumption does not hold (either because the signal and the noise are poorly estimated by the model, or because the noise is not actually Gaussian/Poisson), then the model parameters will be biased and the model less reliable, leading to considerations about whether the assumptions made by the model are valid. Note, however, that there have been recent efforts to fit non-linear models of the input/output function without explicitly assuming distributions of error (Fitzgerald et al., 2011).

Moreover, as with any statistic of brain activity, metrics for predictive power can be artificially inflated. For example, signals that are averaged, smoothed, or otherwise have strong low-frequency power will tend to give larger prediction scores, but may not represent the true relationship between stimuli and brain features. This is one reason to use metrics that are designed with time-series in mind, such as coherence, which does not depend on a particular level of smoothing applied to the data.

#### Estimating the prediction score ceiling

Another useful technique involves determining the highest possible prediction score one would expect given the variability in the data collected. A given *R*^2^ value may be interpreted as “good” or “bad” based off the maximum expected *R*^2^ possible for the dataset. This is called the “noise ceiling” of the data, and it allows one to calculate the percent of *possible* variance explainable by the model, instead of the percent of *total* variance explained by the model.

There is no guaranteed way to calculate the noise ceiling of a model, as it must be estimated from the data at hand. However, there have been attempts at defining principled approaches to doing so. These follow the principle that the recorded neural data is thought to be a combination of “signal” and “noise.”

$$\begin{array}{l} data_{stim\_i}=signal_{stim\_i}+noise \end{array}$$

Note that in this case, only the signal component of the data is dependent on a given stimulus.

One may estimate the noise ceiling of a model based off of the signal-to-noise ratio (SNR) of the neural response to repetitions of the same stimulus. In this case, one randomly splits these repetitions into two groups and calculates the mean response to each, theoretically removing the noise component of the response in each group. The statistic of interest (e.g., *R*^2^) is then calculated between each group. This process is repeated many times, and the resulting distribution of model scores can be used to calculate the noise ceiling. This process is explained in more detail in Hsu et al. (2004) (section Choosing and Fitting the Model) and code for performing this is demonstrated in the Jupyter notebooks associate with this manuscript.

It is possible to perform the same approach using *different* stimuli by assuming that signals and noise have particular statistics. For example, the signal can be assumed to be restricted to low frequencies and the noise to have a normal distribution. If these assumptions hold, then it may be possible to estimate the maximum prediction score, but this risks arriving at a conservative estimate of this value due to some parts of the signal being treated as noise and averaged out. It is also important to note that these approaches assume a linear, invariant neural response to the stimulus, and it is more difficult to assess the theoretical maximum prediction score of the non-linear relationship between inputs and outputs (Sahani and Linden, 2003).

#### A note on multiple comparisons

The ability to perform multivariate analyses is both a blessing and a curse. On one hand, one can relate the activity of many stimulus features to a neural signal within a single modeling framework. On the other, this introduces new considerations when controlling for multiple comparisons and statistical inference.

The most notable benefit for multiple comparisons in the encoding/decoding model framework is the fact that input variables are considered jointly, meaning that it is not always necessary to run an independent test for each variable of interest. Instead, the researcher may inspect the pattern of activity across all model coefficients. For example (Holdgraf et al., 2016), fit STRFs when electrocorticography patients heard degraded speech sentences. The authors compared the shape of the receptive field rather than performing inference on individual model coefficients. As such, relatively fewer statistical analyses were carried out by focusing on *patterns* in the receptive field rather than each parameter independently.

While predictive modeling can reduce the number of statistical comparisons by considering the joint pattern of coefficients across features, it also introduces new challenges for statistical comparisons. For example, natural stimuli offer an opportunity to investigate the relationship between neural activity and many different sets of features (e.g., spectrotemporal features, articulatory features, and words; de Heer et al., 2017). As new features are used to fit models, there is an increased likelihood of a type 1 error. In these cases, it is crucial to define well-formulated hypotheses *before* fitting models with many different input features. Alternatively, one may use an encoding/decoding framework as an exploratory analysis step for the purpose of generating new hypotheses about the representation of stimulus features in the brain. These should then be confirmed on held-out data that has not yet been analyzed, or by follow-up experiments that are designed to test the hypotheses generated from the exploratory step. Ultimately it should be emphasized that while predictive models consider input features simultaneously, they are not a silver bullet for multiple comparisons problems, especially when performing statistical inference on individual model parameters (Curran-Everett, 2000; Maris and Oostenveld, 2007; Bennett et al., 2009).

Another challenge for multiple comparisons comes with the choice of model and the parameters associated with this model. While this paper focuses on linear models with standard regularization techniques (Ridge regression), there are myriad architectures for linking input and output activity. It is tempting to try several types of encoding/decoding models when exploring data, and researchers should be careful that they are not introducing “experimenter free parameters” that may artificially inflate their Type 1 error rate.

Finally, the model itself often also has so-called *hyperparameters* that control the behavior of the model and the kind of structure that it finds in the input data. These hyperparameters have a strong influence on the outcome of the analysis, and should be tuned so that the model performs well on held-out validation data. Importantly, researchers cannot use the same set of data to both tune hyperparameters and test their model. Instead, it is best practice to use an *inner loop* (see above). This reduces the tendency of the model to over fit to training data (Wu et al., 2006; Hastie et al., 2009; Naselaris et al., 2011). If performing statistical inference on model parameters, this should be done *outside* of the inner-loop, after hyperparameters have already been tuned.

## Interpreting the model

If one concludes that the model is capturing an important element of the relationship between brain activity and stimulus properties, one may use it to draw conclusions about the neural process under study. While encoding and decoding models have similar inputs and outputs, they can be interpreted in different, and often complementary ways (Weichwald et al., 2015). The proper method for fitting and interpreting model weights is actively debated, and the reader is urged to consult the current and emerging literature focused on predictive models of brain function (Naselaris et al., 2011; Varoquaux et al., 2016). In the following sections, we describe some challenges and best-practices in using predictive power to make scientific statements about the brain.

### Encoding models

The simplest method for interpreting the results of a model fit is to investigate its weights. In a linear model, a positive weight for a given feature means that higher values of that feature correspond to higher values in the neural signal (they are correlated), a negative weight suggests that increases in the feature values are related to a decrease in the neural signal (they are anti-correlated). If the magnitude of a weight is zero (or very small) it means that fluctuations in the values for that feature will have little effect on the neural signal. As such, investigating the weights amounts to describing the features that a particular neural signal will respond to, presumably because that feature (or one like it) is represented within the neural information at that region of the processing hierarchy. Note that the values of the different features have to be appropriately normalized during model training so that differences in the scale of features does not influence the magnitude of feature weights. This is typically done by z-scoring the values of each feature separately by subtracting its mean and dividing by its standard deviation.

If stimulus features have been chosen such that they have an interpretable meaning, then it is straightforward to assess meaning to the weight of each feature. In addition, if the features have a natural ordering to them (such as increasing frequency bands of a spectrogram, along with multiple time lags for each band), then the pattern of weights represents a receptive field for the neural signal. For example, spectrotemporal receptive fields have been shown to map onto higher-order acoustic features (Woolley et al., 2009) and to increase in complexity as one moves through the auditory pathway (Sen et al., 2001; Miller et al., 2002; Sharpee et al., 2011). This approach has also been used in humans to investigate the tuning properties as one moves across the superior temporal gyrus (Hullett et al., 2016). It is also possible to use statistical methods to find patterns in model coefficients across large regions of cortex. For example (Huth et al., 2012, 2016), fit semantic word models (where each coefficient corresponded to one word) to each voxel in the human cortex. The authors then used Principle Components Analysis to investigate model coefficient covariance across widely distributed regions of the brain, finding consistent axes along which these coefficients covaried with one another.

Finally, another approach toward interpreting encoding models entails comparing model performance across multiple feature representations. For example, in de Heer et al. (2017), the authors investigated the representation of three auditory features (spectral, articulatory, and semantic features) across the cortical surface. They accomplished this by partitioning variance explained by each feature set individually, as well as by joint models incorporating combinations of these features. This enabled them to determine the extent to which each feature is represented across the cortex.

### Decoding models

In a decoding approach, model weights are attached to each neural signal. Higher values for a signal mean that it is more important in predicting the output value of the stimulus/class used in the model. Interpreting the weights of decoding models can be challenging, as weights with a large amplitude do not necessarily mean that the neural signal encodes information about the stimulus (See “section Differences between Encoding and Decoding Models” for a more thorough discussion of this idea). It is important to rely on the statistical reliability of the model weight magnitudes (e.g., low variance across random partitions of data) to extract interpretable features (Reichert et al., 2014).

Finally, it should be noted that in some cases, decoding models are used purely for making optimal predictions about stimulus values. For instance, in neurorehabilitation, decoding models have been used to predict 3D trajectories of a robotic arm for motor substitution (Hochberg et al., 2012). In this case, decoding is approached as an engineering problem, wherein the goal is to obtain the highest decoding predictions and interpreting model weights is of less importance.

### General comments on interpretation

It is possible to use the predictive power of either encoding models (e.g., the *R*^2^ of a model) or decoding models (e.g., the AUC calculated from an ROC curve) to make statements about the nature of stimulus feature representations in the brain. For example, if two models are fit on the same neural data, each with a different set of input features, one may compare the variance explained in the testing data by each model. By fitting multiple models, each with a different feature representation, and comparing their relative prediction scores, one may investigate the extent to which each of these feature representations are a “good” description of the neural response (Huth et al., 2016). However, comparing models with different types or numbers of features is not straightforward, as there are often relationships between the features used in each model, as well as difference in the number of parameters used. In this case, a variance partitioning approach can also be used to distinguish the variance exclusively explained by two (or more) models from the one exclusively explained by one and not the other. This is done by comparing the prediction scores of each model separately, as well as a joint model that includes all possible parameters (Lescroart et al., 2015; de Heer et al., 2017).

It is also possible to investigate the weights and predictive power across models trained in different regions of the brain to investigate how the relationship between stimulus features and brain activity varies across cortex. By plotting a model's predictive power as a function of its neural location, one may construct a tuning map that shows which brain regions are well-predicted by a set of features (Huth et al., 2016). Moreover, by summarizing receptive fields by the feature value that elicits the largest response in brain activity, and plotting the “preferred feature” for each region of the brain, one may construct a *tuning map* that describes how the neural response within a particular set of features is distributed in the brain (Moerel et al., 2013; Hullett et al., 2016; Huth et al., 2016).

By choosing the right representations of features to include in the model, it may be possible to reliably predict all of the variability in brain activity that is dependent on the controlled experimental parameters. Note that the activity that arises from non-experimental factors, e.g., from internal states not controlled in the experiment or from neural and measurement noise, cannot be predicted. This goal requires special considerations for choosing stimuli and experimental design, which will be discussed in the final section.

## Differences between encoding and decoding models

### Differences in terminology and causality

While it is tempting to treat encoding and decoding models as two sides of the same coin, there are important differences between them in an experimental context. Encoding and decoding models have different assumptions about the direction of causality that may influence the possible interpretations of the model depending on the experiment being conducted.

Encoding models are often called *Forward* models, reflecting the direction of time from stimulus to neural activity. Conversely, decoding models are often called *Backward* or *Inverse* models, as they move “backwards” in time in a traditional sensory experiment (Thirion et al., 2006; Crosse et al., 2016). However, it should be noted that this is not always the case, as sometimes a decoded value (e.g., a movement) is actually driven by neural activity. For this reason we prefer the more specific terminology of *encoding* and *decoding*.

The nature of the experiment may also influence the terminology employed. For example, in an experimental paradigm in which stimuli in the world give rise to recorded brain activity (e.g., an experiment where subjects listen to speech), an encoding model naturally models the direction of causality from stimuli to brain activity. As such, it is called a *causal* model. On the other hand, in this experiment a decoding model operates in the opposite direction, inferring properties of the world from the neural activity. This is often called an *acausal* model.

The importance of specifying the direction of causality, and accounting for this in model choice and interpretation, is discussed in greater detail in Weichwald et al. (2015). The following sections describe some important considerations.

### Differences in regression

It is possible for decoding models to be constructed with a regression framework, similarly to how encoding models operate. For example, in Mesgarani and Chang (2012) and Pasley et al. (2012), the experimenters fit one model for each stimulus feature being decoded. This amounts to simply reversing the terms in the standard regression equations:

$$\begin{array}{l} weights_{encoding}={\left(X^{T}X\right)}^{-1}X^{T}y\\ weights_{decoding}={\left(Y^{T}Y\right)}^{-1}Y^{T}x \end{array}$$

It is tempting in this case to collect the coefficients of each decoding model and interpret this as if they came from an encoding model. However, it's important to note that a primary role of regression is to account for correlations between input features when estimating model coefficients. As explained in detail in Weichwald et al. (2015), if a stimulus feature *X*_*i*_ causally influences a neural feature *Y*_*i*_, , and if the stimulus feature *X*_*i*_ is *correlated* with another stimulus feature *X*_*j*_ (for example, if they share correlated noise, or if the stimulus features are naturally correlated), the decoder will give significant weights for both *X*_*i*_ and *X*_*j*_, even though it is only *X*_*i*_ that influences the neural signal. This fact has important implications in the interpretation of model weights.

**Figure 5:**
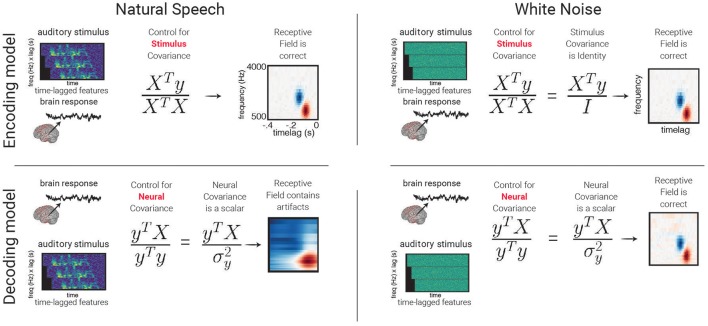
Comparing encoding and decoding weights. An example of how using an encoding or a decoding framework can influence model results. In this case, we attempt to find the relationship between spectral features of sound and the neural activity. **Left, upper**: Using an encoding model, we naturally control for the covariance of the stimulus. Because the stimulus is natural speech, the correlations between lags and frequencies are accounted for, and the correct receptive field is recovered. **Left, lower**: In a decoding model, the X and y terms are reversed, and we instead control for the covariance of the neural activity. Because we have only one neural channel, the covariance term becomes a scalar (the variance of the neural signal). The decoded model weights are smeared in time and frequency. This is because of correlations that exist between these stimulus features. **Right, upper/lower**: The same approach applied to white noise stimuli instead of natural speech. In this case, there are no correlations between stimulus features, and so the covariance matrix becomes the identity matrix, making the receptive field in the encoding and decoding approach roughly the same. While this example is shown for receptive field modeling, the same caveat applies to any modeling framework where there are correlations between either inputs or outputs.

Consider the case of receptive field modeling, in which auditory stimuli are presented to the individual, and a model is fit to uncover the spectral features to which the neural activity responds. In the encoding model, correlations between stimulus features are explicitly accounted for (*X*^*T*^*X*), while in the decoding model, correlations between the *neural* features are accounted for (*Y*^*T*^*Y*). While it is possible to retrieve a receptive field using a decoding paradigm (e.g., by fitting one decoding model for each frequency/time-lag and collecting coefficients into a STRF), correlations in the stimulus features will skew the distribution of model coefficients. This might result in a STRF that is smoothed over a local region in delay/frequency. An encoding model should (theoretically) take these stimulus correlations into account, and only assign non-zero coefficient values to the proper features (see Figure 5). In this case it is important to consider the regularizer used in fitting the model, as there are differences in how regularization techniques distribute model weights with correlated features (Mesgarani et al., 2009).

### Differences in classification

The direction of causality also has important implications in the interpretation of classifiers. It is common to fit a classifier that predicts a stimulus type or neural state using neural features as inputs. In this case, it is tempting to interpret the magnitude of each weight as the extent to which that neural signal carries information about the state being decoded. However, this may not be the case. Following the logic above, if a neural signal with *no* true response to a stimulus is correlated with a neural signal that *does* respond to a stimulus, the classifier may (mistakenly) give positive magnitude to each. As such, one must exercise caution when making inferences about the importance of neural signals using model coefficients in an *Acausal* decoding model (Mesgarani et al., 2009; Haufe et al., 2014).

For example, monitoring the activity of brain regions *not* involved in representing stimulus features but instead reflecting some internal state (e.g., attention) may improve the quality of the decoder performance if attention is correlated with stimulus presentation. Such an effect would be due to the multivariate nature of the decoding model and could, in principle, be detected with additional univariate analyses. This is true of many decoding models, and may cause erroneous conclusions about an electrode's role in processing sensory features. However, as explained in Weichwald et al. (2015), the potential difficulty for causal interpretations in decoding approaches does not negate their usefulness: encoding and decoding models can be used in a complementary fashion to describe potential causal relationships between stimulus and corresponding neural activity in different brain regions.

## Experimental design

While much of this paper has covered the technical and data analytic side of predictive modeling, it is also important to design experiments with predictive models in mind. Fitting encoding and decoding models effectively requires particular considerations for the experimental manipulations and stimulus choices. We will discuss some of these topics below.

### Task design

While traditional experiments manipulate a limited number of independent variables between conditions, the strength of predictive modeling lies in using complex stimuli with many potential features of interest being presented continuously and overlapping in time. This has the added benefit that complex stimuli are generally closer to the “real world” of human experience. This adds to the experiment's *external validity*, which can be difficult to achieve with traditional experimental designs (Campbell and Stanley, 2015).

The simplest task for an encoding model framework is to ask the subject to passively perceive a stimulus presented to them. For example, Huth et al. asked subjects to listen to series of stories told in the podcast *The Moth* (Huth et al., 2016). There was no explicit behavioral manipulation required of the subjects, other than attending to the stories. Using semantic features extracted from the audio, as well as BOLD activity collected with fMRI, the researchers were able to build encoding models that described how semantic categories drove the activity across wide regions of the cortex.

The use of complex stimuli does not preclude performing experimental manipulation. For example, Holdgraf et al. (2016) presented a natural speech stimulus to ECoG subjects, who were asked to passively listen to the sounds. These sentences came in triplets following a *degraded -*> *clean -*> *degraded* structure. By presenting the same degraded speech stimulus *before* and *after* the presentation of a non-degraded version of the sentence, the experimenters manipulated the independent variable of comprehension, and tested its effect on the neural response to multiple speech features.

It is also possible to ask subjects to actively engage in the task to influence how their sensory cortex interacts with the stimuli. Mesgarani et al. used a decoding paradigm to predict the spectrogram of speech that elicited a pattern of neural activity (Mesgarani and Chang, 2012). They asked the subject to attend to one of two natural speech streams, the classic cocktail party effect. Thus, they experimentally manipulated the subject's attention, while the natural speech stimuli were kept the same. They compared the decoded spectrogram as a function of which speaker the subject was attending to, suggesting that attention modulates the cortical response to spectro-temporal features.

### Stimulus construction

Choosing the proper stimuli is a crucial step in order to properly construct predictive models. A model's ability to relate stimulus features to brain activity is only as good as the data on which it is trained. For a model to be interpretable, it must be fit with a rich set of possible feature combinations that cover the stimulus statistics that are typical for the individual under study, and for the feature representations of interest. For example, it is difficult to make statements about how the brain responds to semantic information if the stimuli presented do not broadly cover semantic space.

There are many stimulus sets that are commonly used in predictive modeling of the auditory system. For example, the TIMIT corpus is a collection of spoken English sentences that are designed to cover a broad range of acoustic and linguistic features (Zue et al., 1990). This may be appropriate for studying lower-order auditory processes, though it is unclear whether stimuli such as these are useful for more abstract semantic processes, as the sentences do not follow any high-level narrative. Efforts have been made to construct more semantically rich stimuli (e.g., Huth et al., 2016), though it is difficult to properly tag a stimulus with the proper timing of linguistic features (e.g., phoneme and word onsets). A database with many types of linguistic/auditory stimuli can be found at *catalog.ldc.upenn.edu*.

### How much data to collect?

The short answer to this question is always “as much as you possibly can.” However, in practice many studies are time-limited in their ability to collect large quantities of data. One should take care to include enough stimuli such that the model has the right amount of data to make predictions on test set data. It is not possible to know exactly how much data is needed as this depends on both the number of parameters in the model as well as the noise in the signal being predicted. However, it is possible to estimate the amount of training samples required to achieve a reasonable predictive score given further assumptions about the complexity of the model and the expected noise variance (similar to traditional statistical power estimation).

Ideally, one should conduct pilot studies in order to determine the minimum number of trials, time-points, and other experimental manipulations required to model the relationship between inputs/outputs to some degree of desired accuracy. It is useful to plot a model's predictive score on testing data as a function of the number of data points included in fitting the model, this is called a *Learning Curve*. At some point, increasing the amount of data in the model fit will no longer result in an improvement in prediction scores. One should collect *at least* enough data such that predictive scores remain stable as more data is added. For insight into what is meant by “stable,” see the simulation performed by Willmore and Smyth on a spiking neuron. These authors showed the shape of the reconstruction error curve for a number of fitting procedures and as a function of the number of stimulus presentations, finding that error decreases as the number of presentations goes up, and eventually bottoms-out (Willmore and Smyth, 2003, Figure 5).

Finally, it is also advised to include multiple repetitions of stimuli that will be used purely for validating the model. This has two substantial benefits. First, having multiple instances of the brain's response to the same stimulus makes it easier to estimate the ceiling on model performance (see section Metrics for Regression Prediction Scores). Second, if these repetitions happen at different points throughout the experiment, it is possible to use them to assess the degree of *stationarity* in the neural response. Most models assume that the relationship between the stimulus features and the brain activity will be stable over time. This is often not the case as brains are inherently plastic (e.g., Meyer et al., 2014; Holdgraf et al., 2016), and may change their responsiveness to stimuli based on experimental manipulations or broader changes such as levels of internal or external attention. Recording the neural response to the same stimulus throughout the experiment provides a metric of whether the assumption of stationarity holds.

## Conclusions

Predictive modeling allows researchers to relate neural activity to complex and naturalistic stimuli in the world. Encoding models provide an objective methodology to determine the ability of different feature representations to account for variability in the neural response. Decoding models play a complementary role to encoding models, and allow for the reconstruction of stimuli from ensembles of neural activity, opening the door for future advancements in neuroprosthetics. Predictive models have been successfully used to model the neural response of single units (e.g., Theunissen et al., 2001), high-frequency electrode activity (e.g., (Mesgarani and Chang, 2012); Stéphanie (Martin et al., 2014); Stephanie (Martin et al., 2016)), and BOLD responses to low-level stimulus features (Nishimoto et al., 2011). They have also been used to investigate the neural response to higher-level stimulus features (e.g., Çukur et al., 2013; Huth et al., 2016), as well as to investigate how this response changes across time or condition (e.g., Fritz et al., 2003; Meyer et al., 2014; Slee and David, 2015).

There are many caveats that come with a predictive modeling framework, including considerations for feature extraction, model selection, model validation, model interpretation, and experimental design. We have discussed many of these issues in this review and have provided python tutorials to guide the reader in implementing these methods. We urge the reader to examine the citations provided for further details and to follow advances in this field closely as our understanding of its drawbacks and its potential continues to evolve.

## Author contributions

CH: Wrote majority of manuscript, conducted literature review, created jupyter notebooks, oversaw contributions of coauthors. JR: Contributed to writing, assisted literature review, and high-level organization and planning. CM: Contributed to writing, assisted literature review, assisted with organization. SM: Contributed to writing, assisted literature review, assisted with organization. Focused on classification. RK: Assisted with writing and editing. FT: Contributed to writing and high-level organization and planning.

### Conflict of interest statement

The authors declare that the research was conducted in the absence of any commercial or financial relationships that could be construed as a potential conflict of interest.

## Footnotes

^1^https://github.com/choldgraf/paper-encoding_decoding_electrophysiology

^2^**Sidenote on gabors**: A Gabor function is a sinusoidal function windowed with a Gaussian density function (in either 1- or 2-D), and is commonly used to derive stimulus representations in both visual (Kay and Gallant, 2009; Naselaris et al., 2009; Nishimoto et al., 2011; Lescroart et al., 2016), and auditory cortex (Theunissen et al., 2001; Qiu et al., 2003; Santoro et al., 2014). For example, it is possible to create a spectro-temporal representation of sounds by constructing a collection of Gabor wavelets with linearly- or logarithmically-increasing frequencies, filtering the raw sound with each one, then calculating the amplitude envelope of the output of each filter. If the nature of the stimulus is 2-D (e.g., an image, movie, or spectro-temporal representation), a collection of 2-D Gabor wavelets may be created with successive frequencies and orientations (Frye et al., 2016). Gabor functions may also be a particularly efficient means of storing stimulus information, and studies that use a sparse coding framework to model the way that neurons represent information often result in Gabor-like decompositions (Olshausen and Field, 1997).

^3^http://beta.mybinder.org/v2/gh/choldgraf/paper-encoding_decoding_electrophysiology/master?filepath=notebooks/Prediction%20and%20Validation.ipynb
